# Supplementary material for: Caregiver-reported social impacts in down syndrome regression disorder
Source: PLoS One. 2026 Feb 4;21(2):e0342148. doi: 10.1371/journal.pone.0342148 (PMC12871964; doi:10.1371/journal.pone.0342148)
Supplement: S1 File — Survey, Part A. (PDF) [file pone.0342148.s001.pdf]

Down Syndrome Regression Disorder Caregiver Distress Survey

Page 1 of 5: Participant Eligibility

Thank you for your interest in completing our caregiver distress survey on DSRD. The purpose of this study is to elucidate the multifaceted impacts of Down Syndrome Regression Disorder on caregivers and families. The following questions determine whether or not you are eligible to complete the survey. If you have any questions, please feel free to contact our research team at knchow@usc.edu.

Do you have a child or family member who has been diagnosed with Down Syndrome Regression Disorder (DSRD) by a physician?

- ◯ I am unsure if my child/family member meets criteria for a diagnosis of possible or probable DSRD
- ◯ Yes
- ◯ No

The following questions will help you assess whether or not your child/family member might meet consensus criteria for possible or probable DSRD. Please note, these questions do not serve as an assessment or diagnosis by a licensed medical professional.

Did your previously health child/family's neurologic, psychiatric, or mixed symptoms begin over a period of less than 12 weeks?

- ◯ Yes
- ◯ No

Does your child/family member have a movement disorder such as catatonia, bradykinesia, freezing, or gait disturbance? This does not include tics.

- ◯ Yes
- ◯ No

Does your child/family member have any of the symptom categories below?
To meet criteria for possible DSRD, your child/family member must have 2 of these symptom categories.
To meet criteria for probable DSRD, your child/family member must have 5 of these symptom categories.

- ☐ Altered mental status or behavioral dysregulation (anorexia, decreased oral intake, hyperphagia, confusion, disorientation, inappropriate laughter, or encephalopathy)
- ☐ Cognitive decline (apathy, abulia, avolition, memory impairment)
- ☐ Developmental regression with or without autistic features (social withdrawal, loss of previously acquired developmental milestones, inability to perform activities of daily living, stereotypy, rigidity with routines, decreased eye contact, decreased empathy/social engagement)
- ☐ Focal neurologic deficits on examination and/or seizure
- ☐ Insomnia or circadian rhythm disruption not explained by another cause
- ☐ Language deficits (expressive or receptive aphasia, global aphasia, whispered speech, neologisms, echolalia, coprolalia, paralalia)
- ☐ Psychiatric symptoms (anxiety, delusions, hallucinations, derealization, depersonalization, obsessive compulsive tendencies, aggression, agitation)
- ☐ My child/family member has none of these symptoms

Hidden field:

Participants meets symptom cluster criteria from question above

____

Your child/family member might not meet criteria for a diagnosis of possible or probable DSRD.   Please do not fill out the remaining questions.   Thank you so much for your time. We really appreciate your assistance with our research on DSRD!

Are you over the age of 18?	◯ Yes<br>◯ No
Are you generally knowledgeable about your child or family member's diagnosis, symptoms, and treatment plan?	◯ Yes<br>◯ No
Are you interested in participating in this study? Please note, detailed information on the study will be provided on the next page if you are eligible to participate.	◯ Yes<br>◯ No

Unfortunately, you are not eligible to participate in this research study.   Please do not click submit.   Thank you so much for your time. We really appreciate your assistance with our research on DSRD!

Please click the submit button below to continue on to the research information page.

Down Syndrome Regression Disorder Caregiver Distress Survey

Page 2 of 5: Study Information Page

Children's Hospital Los Angeles
RESEARCH INFORMATION SHEET
Investigating Caregiver Distress and Family Impact of an Individual with Down Syndrome Regression Disorder (DSRD)

You are invited to join a research study led by Jonathan Santoro, M.D. from the division of Neurology at Children's Hospital Los Angeles (CHLA).

The purpose of this study is to elucidate the multifaceted impacts of Down Syndrome Regression Disorder on caregivers and families. The investigators are interested in understanding the extent and nature of familial distress in this context, as it is crucial for developing targeted interventions and support systems to alleviate caregiver burden and enhance family resilience. If you volunteer to participate in this study, your participation will last 15 minutes and involve filling out an online survey.

This study involves the use of your personal survey information. The survey will be anonymous, however, so there will be no way for anyone to link your survey responses back to your identity. Some of the questions in the survey may be personal or upsetting. You do not have to answer any questions that you do not feel comfortable answering. You should not expect any direct benefit as a result of participating in this research. This study will help researchers learn more about Down syndrome Regression Disorder (DSRD). By identifying the challenges that caregivers and families face, investigators can better tailor research studies and clinical trials in the future. The data will be published and be available to patients, caregivers, physicians and others in the medical community. The alternative to participation is to not participate.

There are no costs for participating in this study. You will not be paid for your participation in this research study.

The research team will keep your data confidential. None of the information will be disclosed to others, except if necessary to protect your rights or welfare or if required by law (i.e., harm to self or others, reports of certain infectious diseases). Government agencies, such the Department of Health and Human Services, the CHLA Institutional Review Board (IRB) that reviewed this research, and authorized representatives of CHLA may also have access to the study records in order to oversee the study. You will not be identified in any publications of the research results.

Your participation in the study is entirely voluntary. If you choose not to take part in the study or decide to stop your participation in this study at any time, there will be no penalty or loss of benefits to which you are otherwise entitled. Your choice about whether or not to participate will have no effect on your care, services or benefits at Children's Hospital Los Angeles.

If you have questions, concerns, or complaints about the study, or think this research has harmed you, talk to the CHLA research team at (323) 607-3505.

This research is being overseen by the CHLA Institutional Review Board ("IRB"). An IRB is a group of people who perform ethical review of research studies. You may talk to them at (323) 361-2265, or hspp@chla.usc.edu if:
• You have questions, concerns, or complaints that are not being answered by the research team.
• You are not getting answers from the research team.
• You cannot reach the research team.
• You want to talk to someone else about the research.
• You have questions about your rights as a research subject.

Version Date: xxxxxxx
IRB#: xxxxxx

Please click the submit button below to continue on to the survey.

Down Syndrome Regression Disorder Caregiver Distress Survey

Page 3 of 5: PedsQL

Thank you for taking the time to complete this survey. Please note, you may skip any questions that make you feel uncomfortable. You can also decide to stop filling out the survey at any time.

Directions Families of children sometimes have special concerns or difficulties because of the child's health. On the following page is a list of things that might be a problem for you. Please tell us how much of a problem each one has been for you during the past ONE month by choosing:

0 if it is never a problem
1 if it is almost never a problem
2 if it is sometimes a problem
3 if it is often never a problem
4 if it is almost always a problem

There are no right or wrong answers.
If you do not understand a question, please ask for help.

In the past ONE month, as a result of your child's health, how much of a problem have YOU had with...

PHYSICAL FUNCTIONING (problems with...)

	Never (0)	Almost Never (1)	Sometimes (2)	Often (3)	Almost Always (4)
I feel tired during the day	○	○	○	○	○
I feel tired when I wake up in the morning	○	○	○	○	○
I feel too tired to do the things I like to do	○	○	○	○	○
I get headaches	○	○	○	○	○
I feel physically weak	○	○	○	○	○
I feel sick to my stomach	○	○	○	○	○

EMOTIONAL FUNCTIONING (problems with...)

	Never (0)	Almost Never (1)	Sometimes (2)	Often (3)	Almost Always (4)
I feel anxious	○	○	○	○	○
I feel sad	○	○	○	○	○
I feel angry	○	○	○	○	○
I feel frustrated	○	○	○	○	○
I feel helpless or hopeless	○	○	○	○	○

SOCIAL FUNCTIONING (problems with...)

	Never (0)	Almost Never (1)	Sometimes (2)	Often (3)	Almost Always (4)
I feel isolated from others	○	○	○	○	○
I have trouble getting support from others	○	○	○	○	○
It is hard to find time for social activities	○	○	○	○	○
I do not have enough energy for social activities	○	○	○	○	○

COGNITIVE FUNCTIONING (problems with...)

	Never (0)	Almost Never (1)	Sometimes (2)	Often (3)	Almost Always (4)
It is hard for me to keep my attention on things	○	○	○	○	○
It is hard for me to remember what people tell me	○	○	○	○	○
It is hard for me to remember what I just heard	○	○	○	○	○
It is hard for me to think quickly	○	○	○	○	○
I have trouble remembering what I was just thinking	○	○	○	○	○

COMMUNICATION (problems with...)

	Never (0)	Almost Never (1)	Sometimes (2)	Often (3)	Almost Always (4)
I feel that others do not understand my family's situation	○	○	○	○	○
It is hard for me to talk about my child's health with others	○	○	○	○	○
It is hard for me to tell doctors and nurses how I feel	○	○	○	○	○

In the past ONE month, as a result of your child's health, how much of a problem have you had with...

WORRY (problems with...)

	Never (0)	Almost Never (1)	Sometimes (2)	Often (3)	Almost Always (4)
I worry about whether or not my child's medical treatments are working	○	○	○	○	○

I worry about the side effects of my child's medications/medical treatments	○	○	○	○	○
I worry about how others will react to my child's condition	○	○	○	○	○
I worry about how my child's illness is affecting other family members	○	○	○	○	○
I worry about my child's future	○	○	○	○	○

DIRECTIONS Below is a list of things that might be a problem for your family. Please tell us how much of a problem each one has been for your family during the past ONE month.

In the past ONE month, as a result of your child's health, how much of a problem has your family had with...

DAILY ACTIVITIES (problems with...)

	Never (0)	Almost Never (1)	Sometimes (2)	Often (3)	Almost Always (4)
Family activities taking more time and effort	○	○	○	○	○
Difficulty finding time to finish household tasks	○	○	○	○	○
Feeling too tired to finish household tasks	○	○	○	○	○

FAMILY RELATIONSHIPS (problems with...)

	Never (0)	Almost Never (1)	Sometimes (2)	Often (3)	Almost Always (4)
Lack of communication between family members	○	○	○	○	○
Conflicts between family members	○	○	○	○	○
Difficulty making decisions together as a family	○	○	○	○	○
Difficulty solving family problems together	○	○	○	○	○
Stress or tension between family members	○	○	○	○	○

Down Syndrome Regression Disorder Caregiver Distress Survey

Page 4 of 5: Caregiver Assessment

DIRECTIONS The following is a list of statements that reflect how people sometimes feel when taking care of another person. After reading each statement, indicate how often you experience the feelings listed by selecting the option that best corresponds to the frequency of these feelings.

	Never (0)	Almost Never (1)	Sometimes (2)	Often (3)	Almost Always (4)
Do you feel you don't have enough time for yourself?	○	○	○	○	○
Do you feel stressed between caring and meeting other responsibilities?	○	○	○	○	○
Do you feel angry when you are around your relative?	○	○	○	○	○
Do you feel your relative affects your relationship with others in a negative way?	○	○	○	○	○
Do you feel strained when are around your relative?	○	○	○	○	○
Do you feel your health has suffered because of your involvement with your relative?	○	○	○	○	○
Do you feel you don't have as much privacy as you would like, because of your relative?	○	○	○	○	○
Do you feel your social life has suffered because you are caring for your relative?	○	○	○	○	○
Do you feel you have lost control of your life since your relative's illness?	○	○	○	○	○
Do you feel uncertain about what to do about relative?	○	○	○	○	○
Do you feel you should be doing more for your relative?	○	○	○	○	○
Do you feel you could do a better job in caring for your relative?	○	○	○	○	○

Down Syndrome Regression Disorder Caregiver Distress Survey

Page 5 of 5: Down Syndrome Regression Disorder Caregiver Survey

DIRECTIONS Please answer the following questions. Please feel free to use the empty text boxes to elaborate on your responses.

Financial Impact

	Have you or a caregiver had to modify work hours because of your child/relative's DSRD diagnosis?	◯ Yes<br>◯ No
	Have you or a caregiver had to stop working because of your child's/relative's DSRD diagnosis?	◯ Yes<br>◯ No
3	Have you or a caregiver experienced increased financial burden or stress as a result of your child's/relative's DSRD diagnosis? If yes, please elaborate.	◯ Yes ______<br>◯ No
	Have you or a caregiver had to modify your educational or career plans and goals because of your child's/relative's DSRD diagnosis? If yes, please elaborate.	◯ Yes ______<br>◯ No

Housing

1	Have you had to change your housing situation to accommodate your child's/relative's DSRD diagnosis? (e.g., had to move to a house with no stairs due to catatonia) If yes, please elaborate.	◯ Yes ______<br>◯ No
1	Have you had to move closer to your medical care because of your child's/relative's DSRD diagnosis? If yes, please elaborate.	◯ Yes ______<br>◯ No
1	Due to your child's/relative's DSRD symptoms, have you encountered any issues with landlords, housing associations, or similar groups? (e.g., noise complaints) If yes, please elaborate.	◯ Yes ______<br>◯ No
1	Due to your child's/relative's DSRD symptoms, have you had any issues where law enforcement has been called to your home? (e.g., domestic disturbance due to agitation or hallucinations) If yes, please elaborate.	◯ Yes ______<br>◯ No

Sleep

	Have your sleeping arrangements changed because of your child's/relative's DSRD diagnosis?	◯ Yes<br>◯ No

Check all that apply.	☐ Less sleep (total hours)<br>☐ Sleep in shifts so one caregiver is always awake<br>☐ Changed sleep hours to match loved one with DSRD (e.g., later bed time)<br>☐ Other ______
Has your sleep quality been negatively impacted because of your child's/relative's DSRD diagnosis?	◯ Yes<br>◯ No
Check all that apply.	☐ Disrupted sleep (multiple awakenings)<br>☐ Fragmented sleep (sleeping in shorter stints when things are calm)<br>☐ Not feeling rested upon awakening (daytime sleepiness or fatigue)<br>☐ Need to nap during the day<br>☐ Other ______
Siblings	
Does your child/relative with DSRD have siblings?	◯ Yes<br>◯ No
Has your child's/relative's relationship with their sibling(s) been impacted by your child's/relative's DSRD diagnosis? Please elaborate if possible.	◯ Positive ______<br>◯ Negative ______<br>◯ No Change
Has your child's/relative's sibling's mental health been impacted by your child's/relative's DSRD diagnosis?	◯ Developed or worsened mental health condition intercurrently<br>◯ No change
Check all that apply.	☐ Depression<br>☐ Anxiety<br>☐ Post-traumatic stress disorder (PTSD)<br>☐ Bipolar disorder<br>☐ Dysthymia (persistent depressive disorder)<br>☐ Other ______
Have your child's/relative's sibling's life goals been impacted by your child's/relative's DSRD diagnosis? (e.g., educational attainment, career, etc.) If yes, please elaborate.	◯ Yes ______<br>◯ No
Social Networks and Relationships	
Have the quality of your adult friendships been impacted because of your child's/relative's DSRD diagnosis? Please elaborate if possible.	◯ Positive ______<br>◯ Negative ______<br>◯ No Change
Have your social networks been impacted because of your child's/relative's DSRD diagnosis? (i.e. friends, family, local community) Please elaborate if possible.	◯ Positive ______<br>◯ Negative ______<br>◯ No Change
Has your marriage or domestic partnership been impacted because of your child's/relative's DSRD diagnosis? Please elaborate if possible.	◯ Positive ______<br>◯ Negative ______<br>◯ No Change

Has your friend network changed in size because of your child's/relative's DSRD diagnosis? Please elaborate if possible.	◯ Positive ______<br>◯ Negative ______<br>◯ No Change
Before my child's/relative's diagnosis, my primary positive support networks consisted of: (check all that apply)	☐ Friends<br>☐ Family<br>☐ Siblings<br>☐ Church<br>☐ Medical teams<br>☐ Local community<br>☐ Online community - Facebook or Other<br>☐ Local Down Syndrome organizations<br>☐ National Down Syndrome organizations<br>☐ Other: ______
After my child's/relative's diagnosis, my primary positive support networks consists of: (check all that apply)	☐ Friends<br>☐ Family<br>☐ Siblings<br>☐ Church<br>☐ Medical teams<br>☐ Local community<br>☐ Online community - Facebook or Other<br>☐ Local Down Syndrome organizations<br>☐ National Down Syndrome organizations<br>☐ Other: ______
Mental Health	
Have you experienced worsened mental health because of your child's/relative's DSRD diagnosis?	◯ Yes<br>◯ No
Do you feel frustration or anger towards your loved one because of the regression associated with your child's/relative's DSRD diagnosis? If yes, please elaborate.	◯ Yes ______<br>◯ No
Has a caregiver required evaluation by a mental health professional related to your child's/relative's DSRD diagnosis? (check all that apply)	☐ Psychologist<br>☐ Psychiatrist<br>☐ Family therapist<br>☐ Other: ______
Have you developed any of the following mental health conditions after your child's/relative's DSRD diagnosis? (check all that apply)	☐ Depression<br>☐ Anxiety<br>☐ Post-Traumatic Stress Disorder (PTSD)<br>☐ Bipolar Disorder<br>☐ Dysthymia (persistent depressive disorder)<br>☐ Other ______
How was your mental health condition diagnosed?	◯ Mental health professional<br>◯ Self-diagnosis
Do you have fears about your child's/relative's future or the future of your family since the diagnosis of DSRD? If yes, please elaborate.	◯ Yes ______<br>◯ No
Did you have fears/concerns about misdiagnosis before the official diagnosis of DSRD? If yes, please elaborate.	◯ Yes ______<br>◯ No

Did you have fears/concerns about inadequate access to treatment after the diagnosis of DSRD? If yes, please elaborate.	◯ Yes ______<br>◯ No
If you're experiencing mental health symptoms, remember there are resources available to support and help you-reach out today. Here are some online resources:<br>https://www.nimh.nih.gov/health/find-help<br>https://www.mhanational.org/self-help-tools	
Have you or your family experienced any other impacts, positive or negative, because of your child's/relative's DSRD diagnosis?	________________________________
If you have an extra 5 minutes, please complete this OPTIONAL additional mental health questionnaire. This will help us better assess the mental health of caregivers.	◯ Yes, I would like to take the OPTIONAL survey.<br>◯ No - I am finished.
In the last week, have you felt sad?<br>Have you felt upset?<br>Have you felt miserable?<br>Have you felt depressed?	◯ Yes ______<br>◯ No
In the last week, have you felt as if you are in a bad mood?<br>Have you lost your temper?<br>Have you felt as if you want to shout at people?	◯ Yes ______<br>◯ No
In the last week, have you enjoyed the things you've done?<br>Have you had fun?<br>Have you enjoyed yourself?	◯ Yes ______<br>◯ No
In the last week, have you enjoyed talking to people and being with other people?<br>Have you liked having people around you?<br>Have you enjoyed other people's company?	◯ Yes ______<br>◯ No
In the last week, have you made sure you have washed yourself, worn clean clothes, brushed your teeth and combed your hair?<br>Have you taken care of the way you look?<br>Have you looked after your appearance?	◯ Yes ______<br>◯ No
In the last week, have you felt tired during the day?<br>Have you gone to sleep during the day?<br>Have you found it hard to stay awake during the day?	◯ Yes ______<br>◯ No
In the last week, have you cried?	◯ Yes ______<br>◯ No
In the last week, have you been able to pay attention to things like watching TV?<br>Have you been able to concentrate on things (like TV shows?)	◯ Yes ______<br>◯ No

In the last week, have you found it hard to make decisions?<br>Have you found it hard to decide what to wear, or what to do?<br>Have you found it hard to choose between two things?	◯ Yes ______<br>◯ No
In the last week, have you found it hard to sit still?<br>Have you fidgeted when you are sitting down?<br>Have you been moving around a lot, like you can't help it?	◯ Yes ______<br>◯ No
In the last week, have you been eating too little or eating too much?<br>Do people say you should eat more or less?	◯ Yes ______<br>◯ No
In the last week, have you found it hard to get a good night's sleep?<br>Have you found it hard to fall asleep at night?<br>Have you woken up in the middle of the night and found it hard to get back to sleep?<br>Have you woken up too early in the morning?	◯ Yes ______<br>◯ No
In the last week, have you felt that life is not worth living?<br>Have you wished you could die?<br>Have you felt you do not want to go on living?	◯ Yes ______<br>◯ No
In the last week, have you felt as if everything is your fault?<br>Have you felt as if people blame you for things?<br>Have you felt that things happen because of you?	◯ Yes ______<br>◯ No
In the last week, have you felt that other people are looking at you, talking about you, or laughing at you?<br>Have you worried about what other people think of you?	◯ Yes ______<br>◯ No
In the last week, have you become very upset if someone says you have done something wrong or you have made a mistake?<br>Do you feel sad if someone disagrees with you or argues with you?<br>Do you feel like crying if someone disagrees with you or argues with you?	◯ Yes ______<br>◯ No
In the last week, have you felt worried?<br>Have you felt nervous?<br>Have you felt tense/wound up/on edge?	◯ Yes ______<br>◯ No
In the last week, have you thought that bad things keep happening to you?<br>Have you felt that nothing nice ever happens to you anymore?	◯ Yes ______<br>◯ No

In the last week, have you felt happy when something good happened?
If someone gave you a nice present, would that make you happy?

◯ Yes ______
◯ No
