# Supplementary material for: Caregiver-reported social impacts in down syndrome regression disorder
Source: PLoS One. 2026 Feb 4;21(2):e0342148. doi: 10.1371/journal.pone.0342148 (PMC12871964; doi:10.1371/journal.pone.0342148)
Supplement: S2 File — Survey, Part B. (PDF) [file pone.0342148.s002.pdf]

Survey Instructions: Please answer the following questions. You may skip questions you do not want to answer.

Thank you!

Demographic Questions: Complete the following questions based on how you identify.

1. Current Age (years): ___________
2. Sex at birth
    a. Male
    b. Female
    c. Prefer not to answer
3. Race
    a. White
    b. Black of African American
    c. Asian
    d. Native Hawaiian or Pacific Islander
    e. American Indian or Alaskan Native
    f. Other
    g. Prefer not to answer
4. Ethnicity
    a. Hispanic or Latino
    b. Not Hispanic or Latino
    c. Prefer not to answer
5. Relationship to the individual with DSRD or neurological condition
    a. Mother
    b. Father
    c. Aunt/Uncle
    d. Cousin
    e. Guardian
    f. Other
6. Primary language at home
    a. English
    b. Other

Demographic Questions: Complete the following questions based on the individual with DSRD or neurologic condition.

1. Age of individual at the time of survey (years): _______
2. Sex at birth

    a. Male
    b. Female
    c. Prefer not to answer
3. Race
    a. White
    b. Black of African American
    c. Asian
    d. Native Hawaiian or Pacific Islander
    e. American Indian or Alaskan Native
    f. Other
    g. Prefer not to answer
4. Ethnicity
    a. Hispanic or Latino
    b. Not Hispanic or Latino
    c. Prefer not to answer
5. Duration of symptoms (time since diagnosis in years): ____________
6. For individuals who do NOT have DSRD, what type of neurological diagnosis does your loved one have:
    a. Atlanto-axial instability
    b. Autism spectrum disorder
    c. Cerebrovascular disease or stroke (moyamoya disease or other)
    d. Concussion or traumatic brain injury
    e. Dementia (or Alzheimer's disease)
    f. Epilepsy (or infantile spasms)
    g. Not applicable (loved one has DSRD)
