# Supplementary material for: Caregiver-reported social impacts in down syndrome regression disorder
Source: PLoS One. 2026 Feb 4;21(2):e0342148. doi: 10.1371/journal.pone.0342148 (PMC12871964; doi:10.1371/journal.pone.0342148)
Supplement: Attachment — Submitted filename: Author Responses R1.docx [file pone.0342148.s004.docx]

December 9^th^, 2025

Dear Editors of *PLoS One*,

Thank you for the opportunity to review our manuscript. The authors sincerely appreciate the time and effort made in reviewing the manuscript and providing helpful feedback. The authors apologize for much of the confusion around the methodology of the manuscript and believe that the critiques provided by reviewers have helped rectify much of this. We are hopeful that the revised manuscript will be suitable for publication with the responses provided.

Sincerely,

Jonathan D. Santoro, MD

Reviewer #1: The authors deserve credit for highlighting the challenges faced by caregivers of people with DSRD. This is an important contribution of the article.

In the section entitled 'Limitations', the authors point out several weaknesses in the methodology, including the possibility of recall and selection biases, the lack of recording of demographic factors such as education and socioeconomic status, and the lack of longitudinal follow-up. However, I have a few comments.

| **Reviewer Comment** | **Author Responses** |
| --- | --- |
| Participants were recruited from a neurology clinic and a Facebook support group for DSRD caregivers. However, the authors do not specify how many participants came from each group. It can be assumed that the DSRD diagnosis in the neurology clinic group is correct. This is more difficult to assume for the other group, as the survey contains a number of difficult medical terms, and participants were not provided with guidance by one of the researchers when completing it. This could be a potential source of inaccuracy. | Thank you for identifying this clarity issue. The authors have added this information to the results section (first paragraph) as follows:  “*In the DSRD cohort, 141 (61.8%) were recruited from clinics and 87 (38.2%) were recruited from online groups compared to 94 (68.6%) and 43 (31.4%) in the DSN cohort, respectively (p=0.19)*.”  The similarity between the groups and the lack of statistical significance between the percentages recruited from different environments would diminish some of these concerns although it remains possible that difficulty with medical terminology could influence responses. |
| It is also unclear whether the survey was validated in advance, which could also be a potential source of inaccuracy. | Thank you for identifying this clarity issue. The authors validated the survey for readability, comprehension and time to complete. The authors have added this clarifying statement to the methods section as follows:  “*Prior to release of the survey to prospective participants, the survey was validated for readability, comprehension and time to complete by a group of 10 caregivers. Feedback was collected in an open format and modifications for language, user-experience, and organization were made based on the feedback of these caregivers*.”  However, we also acknowledge that the survey has not yet undergone full psychometric validation (e.g., factor structure and test–retest reliability in independent samples). We now explicitly note this in the Survey and Qualitative Analysis subsection and reiterate it as a limitation in the Discussion.  The authors have thus added the following statement to the limitations section of the discussion:  “*In addition, the tool was partially validated by caregivers for readability and clarity although the authors acknowledge that the survey has not yet undergone full psychometric validation (e.g., factor structure and test–retest reliability in independent samples).”* |
| The authors state that they collected demographic and clinical information via the survey. However, these questions are not included in Appendix A. | Thank you for this comment. The authors have amended the Appendix to include this information with the full survey. This appears to have been a Redcap formatting issue for which the authors apologize. |
| Table 2 lists the mean age, gender, ethnicity, race, and mean duration of symptoms. However, in the Results section, no distinction is made according to ethnicity or race regarding the impact on caregivers. Thus, the significance of mentioning this in Table 2 is unclear. | Thank you for this comment. The authors note that as there was no statistical significance between the groups with regards to race (*p*=0.78) and ethnicity (*p*=0.37). The data reported in the results section was already adjusted for race, ethnicity, and age. This has now been explicitly stated in the methods section as follows:  *“A p-value of less than 0.05 was considered to be statistically significant, with values trending toward this threshold noted for further consideration. All p-values are reflected of adjusted analysis to control for age, ethnicity and race.”*  The authors apologize for this confusion. |
| I also miss the mention of how many of the DSRD group have already been treated and what the outcome was. It would have been interesting to analyze whether that would make a difference to the QOL of the caregivers. | Thank you for this comment. This information was unfortunately not collected in the original survey. As it can take several years to acquire clinical stability and improvements and there is wide heterogeneity in treatments offered regionally, this response could have been heavily skewed and may lead to over-interpretation of “treatment” in this context.  The authors agree that this is a logical next step as it would be presumed that quality of life would improve over time.  Given the impact of this, the authors have mentioned this explicitly in the limitations section of the discussion as follows:  “*In addition, treatment data was not collected due to the lack of ability to verify interventions, timing of interventions, and the lack of ability to adjust for regional therapeutic offering differences. The authors believe that successful treatment of DSRD would improve QOL and this represents a future area of study for the team*.” |
| It is also never mentioned who the caregiver is who completes the survey. | Thank you for identifying this clarity issue. The authors have added this information to the results section as follows:  “*Those completing the survey were most often mothers (166 (72.8%) in DSRD v. 106 (77.4%) in DSN), fathers (43 (18.9%) in DSRD v. 22 (16.1%) in DSN), or guardians (11 (4.8%) in DSRD v. 9 (6.7%) in DSN) (p=0.61)*.” |
| I have another question about Table 2. The mean duration of symptoms in the DSN group is 4.59 years. This surprises me because the authors describe these individuals as young people with ASD, epilepsy, or moyamoya vasculopathy. | Thank you for this comment. The authors note that the diagnosis of autism spectrum disorder in individuals with Down syndrome is heterogeneous and ranges from 3-8 years of age. Both epilepsy and moyamoya vasculopathy are bimodal in their distribution and can happen early or later in childhood. The duration of symptoms for the DSN group corresponds to the years *since* diagnosis of this condition, not the age at which it was diagnosed. Table 2 now has additional information regarding this in the legend to improve clarity as follows:  *“α: indicates years since the diagnosis of this condition”*  It is possible that individuals who have an onset of disease at a younger age could be more or less difficult to handle as a caregiver. This is a possible interpretation of the data as well although there is no literature to support this is an issue in individuals with Down syndrome. Unfortunately, there are no other perfectly matched conditions affecting individuals with DS in the late teen years for which to compare DSRD.  The authors have performed a separate analysis to confirm this hypothesis which is not listed in the results section as follows:  “*Caregiver distress did not worsen with increasing age in either cohort. In the DSRD group, age was not associated with higher odds of endorsing worsening caregiver outcomes (per 1-year increase: OR 1.01; 95% CI 0.98–1.04; p = 0.58). Similarly, in the DSN group, age was not associated with worsening caregiver outcomes (per 1-year increase: OR 0.99; 95% CI 0.95–1.03; p = 0.66). Results were unchanged after adjustment for sex and symptom duration (DSRD adjusted OR 1.00, 95% CI 0.97–1.03, p = 0.91; DSN adjusted OR 0.98, 95% CI 0.94–1.02, p = 0.31*).” |
| The text does not specify how many people in the DSN group have each diagnosis. | Thank you for identifying this clarity issue. The authors have added this information to Table 2. |
| Although the DSN group is younger on average (mean age of 16.05 years), I find it strange that the mean symptom duration is only 4.59 years. A diagnosis of ASD and epilepsy in children is often made at a younger age. Perhaps the authors should verify this. I also wonder how meaningful the comparison between the DSRD and DSN groups is. | Thank you for this comment. As noted above, the reviewer is correct in identifying this theme. The authors note that the diagnosis of autism spectrum disorder in individuals with Down syndrome is heterogeneous and ranges from 3-8 years of age. Both epilepsy and moyamoya vasculopathy are bimodal in their distribution and can happen early or later in childhood. In addition, atlanto-axial instability is often diagnosed in the teen years. These data points are subjectively reported by family members which could introduce a recall bias, as noted in the discussion’s limitations section.  A challenge with this cohort is that responses were anonymous to the research team, making verification of diagnoses not possible. This has been made explicit in the limitations section of the discussion:  *“Additionally, the DSN comparison group was heterogeneous, encompassing various neurologic conditions, which may obscure disorder-specific effects and limit direct comparisons. This is further complicated by this study’s lack of ability to clinically confirm diagnoses.”.*  As noted in the methods, this was specifically detailed to those completing the survey although was not verifiable:  *“In the DSN group, the diagnosis of the neurological condition was based solely on caregiver self-report, and diagnosis by a physician was required in all cases.”*  The authors agree that the meaningfulness of comparison of DSRD to DSN is not perfect but represented a reasonable comparator of medical complexity in the same underlying disease state. It allows interpretation to not simply be related to “just Down syndrome” effect.  As noted above, the authors have added an additional analysis to demonstrate that age did not influence caregiver responses:  “*Caregiver distress did not worsen with increasing age in either cohort. In the DSRD group, age was not associated with higher odds of endorsing worsening caregiver outcomes (per 1-year increase: OR 1.01; 95% CI 0.98–1.04; p = 0.58). Similarly, in the DSN group, age was not associated with worsening caregiver outcomes (per 1-year increase: OR 0.99; 95% CI 0.95–1.03; p = 0.66). Results were unchanged after adjustment for sex and symptom duration (DSRD adjusted OR 1.00, 95% CI 0.97–1.03, p = 0.91; DSN adjusted OR 0.98, 95% CI 0.94–1.02, p = 0.31)*.” |
| The social media quotes are both instructive and moving. | Thank you for this comment. While not typical to include for a study like this the authors agreed that this was important to highlight. |
| Despite its methodological limitations, the article demonstrates that caring for someone with DSDR significantly impacts the social lives of caregivers. However, I have difficulty with the word "permanent" when it comes to social ties. I am not a native English speaker, so perhaps I have misunderstood the meaning of "permanent." To me, it means "lasting forever," which this cross-sectional study with imperfect methodology cannot demonstrate. Perhaps the authors mean something else; I would like to know what that is. | Thank you for this comment. The authors agree that the language of “permanent” is a bit challenging to interpret. The authors agree that this language can be confusing but is designed to reflect a permanence related to the condition. For instance, if there where no changes to a patients condition, would the social network improve. If the answer to this question would be “no” then this would be defined as permanent in this study.  The authors attempted to try different “less permanent” language in the validation trial but caregivers who were evaluating the survey advised that this would be the most easily interpreted for those completing the survey.  The authors appreciate this feedback and will workshop this for future survey-based studies. Taking a more international English language approach may be beneficial for future studies given the reach of DSRD. The following has been added to the limitations section to help clarify this to some extent:  “*In addition, while the thematic coding process was consensus-based and methodologically rigorous, qualitative analysis remains inherently interpretive and may not fully capture the complexity of caregiver experiences.* *Further, the narrower nature of the coding may have oversimplified themes or omitted other areas of social support or strain. In addition, some language such as the use of the term “permanent” or “loss of community participation” can be hard to interpret both for those completing the survey and the researchers coding the data*.” |
| *Minor Remarks* | |
| It appears that the text on lines 116-117 is repeated on lines 118-119. | Thank you for this comment. The authors have removed duplicate lines in the text. |
| Line 179 states: "The DSRD and DSN objective responses are summarized in Table 4." Does "objective responses" mean the same as "descriptive quotations," as written on line 183? If so, it would be better to replace "objective responses" with "descriptive quotations" in the sentence. | Thank you for identifying this clarity issue. The authors have amended this to be consistent and this line now reads “qualitative responses” as follows:  “*The DSRD and DSN qualitative responses are summarized in* ***Table 3***”  The authors have also amended the quotations section at the end of the results section to read as follows:  “*A table of representative descriptive quotations illustrating these themes and providing a deeper qualitative perspective is included in* ***Table 4****.”* |
| The figures are difficult to read. | The authors apologize for this issue. We have increased the dpi to increase clarity and readability. |

Reviewer #2: Impact of Down Syndrome Regression Disorder on Caregiver Social Networks. The topics of this paper are interesting, though well known. The structure and content must be revised, and results have to be better explained by authors before to be reconsidered.

| **Reviewer Comment** | **Author Responses** |
| --- | --- |
| Title, clarify that is a case study on Children's Hospital Los Angeles and period under study. | We appreciate this suggestion. To provide clearer context while keeping the title concise, we have revised it to:  “*Impact of Down Syndrome Regression Disorder on Caregiver Social Networks: A Cross-sectional Study at a Tertiary Pediatric Center in the United States*.”  The Abstract and Methods now explicitly state that participants were recruited from Children’s Hospital Los Angeles and a DSRD Facebook support group between July 6 and July 28, 2024 as follows:  “*This study had two separate groups of participants: 1) DSRD Cohort: This group consisted of caregivers of individuals with a confirmed or probable diagnosis of DSRD. They were recruited through a neurology clinic at Children's Hospital Los Angeles and a private Facebook support group for DSRD caregivers. The recruitment period lasted 3 weeks (July 6, 2024, to July 28, 2024). 2) DSN Cohort: This group was composed of caregivers of individuals with Down syndrome who also had other active neurological conditions, such as moyamoya vasculopathy, epilepsy, or autism spectrum disorder.*” |
| Abstract has to clarify the goal, and specific health and social implications to face DSRD. | Thank you for identifying this clarity issue. The authors have amended the last sentence of the background section to better clarify this. This now reads as follows:  *“The goal of this study was to evaluate the impact of DSRD on caregiver social networks by comparing their experiences to those of caregivers of individuals with Down syndrome and other neurological disorders (DSN).”* |
| Introduction has to better clarify the research questions of this study, indicating the gap presents in literature that this study endeavors to cover, and provide more theoretical background. Clarify differences with this study cited (Caregiver burden and familial impact in Down Syndrome Regression Disorder. Orphanet J Rare Dis 20, 126).  After that authors can focus on the topics of this study to provide a correct analysis for fruitful discussion. (Please refer to the suggested readings, all of which should be thoroughly reviewed and incorporated into your text.). | Thank you for identifying this clarity issue. The authors now have revised the introduction to clearly describe existing evidence on caregiver burden in DS and other neurological conditions, including our prior study of caregiver burden in DSRD (Chow et al., Orphanet J Rare Dis 2025), and also highlight the specific gap regarding systematic characterization of caregiver *social networks* in DSRD compared with other DS-related neurologic conditions. These changes now read as follows:  “*Regression results in a profound loss of ability to engage in activities of daily living, to the point where most individuals become dependent on their caregivers. This can cause caregivers to experience sleep disturbances, financial distress, and negative impacts on family dynamics and social networks, among other challenges. The time-intensive nature of care demands means that individuals with DSRD require significantly more support than they did prior to their diagnosis, with potential cascading negative effects on caregivers and their social networks.* *While previously published literature has identified caregiver burden, dedicated exploration of the impact on social networks of caregivers is unknown*.”  We then explicitly state our primary research questions: (1) How does caregiving for an individual with DSRD affect caregiver social networks, adult friendships, marital/partner relationships, and social network size? and (2) How do these impacts compare with those experienced by caregivers of individuals with DS and other neurological disorders (DSN)? This section now reads as follows:  *“The purpose of this study is to elucidate the impact of DSRD on caregivers’ social networks and to determine if care needs specific to DSRD are greater than individuals with DS alone. Specifically, it aims to compare changes in social networks for caregivers of individuals with DSRD to those with DS and other neurological disorders (DSN). This study specifically evaluated changes in the quality of adult friendships, impact on social networks, impact on spouse/partner, and social network size.”*  The authors have added the suggested reading to the introduction as well in several locations. This was very beneficial in providing an additional background for the readers. These referenced statements are as follows:  “*With appropriate identification, interventional strategies have been identified in DS populations, making the need for identification significant*.”  “The purpose of this study is to elucidate the impact of DSRD on caregivers’ social networks *and to determine if care needs specific to DSRD are greater than individuals with DS alone*.” |
| *Methods* | |
| The section of Materials and methods must be re-structured with the following three sections and same order:  • Sample and data  • Measures of variables  • Data analysis procedure. | Thank you for identifying this clarity issue. The authors have re-structured the methods section as requested by the review. The authors have also removed sub-sections of the methods as requested below. |
| Insert a flow chart to clarify the logic of the study design. | Thank you for this comment. The authors agree that a flow diagram would be beneficial for this study to improve clarity on responses for the qualitative aspects. The authors have added this as a new “Figure 1”. Please note the prior Figures 1 and 2 are now Figures 2 and 3, respectively. |
| Authors have to avoid a lot of subheadings that create fragmentation and confusion. If necessary, you can use bullet points (same comments for section of results and all sections). | Thank you for this comment. As noted above, these subheadings have been removed and where necessary, are consolidated. We agree that this improves the clarity and readability of the manuscript. |
| *Results* | |
| The study employed a cross-sectional methodology, capturing caregiver experiences at a single point in time. This design limits the ability to establish causality or to track how caregiver social networks evolve over the course of the disorder. DSRD is often characterized by acute or subacute regression, and the trajectory of caregiver distress may fluctuate depending on disease progression, access to interventions, or changes in family circumstances. A longitudinal approach would have provided richer insights into the dynamic nature of social withdrawal and the potential for recovery or adaptation. | Thank you for this comment. The authors acknowledge that the cross-sectional design of this study merely establishes that there is social network changes in caregivers of individuals with DSRD compared to other neurological disorders affecting individuals with Down syndrome. Longitudinal assessment would be highly beneficial as it would track the dynamic changes of caregiver burden and social network changes over time, particularly with the initiation of therapy. This is longitudinal study is already underway but will take several year to complete.  The author have added the following statement to the limitations section of the discussion:  “*In addition, this study is limited by its cross-sectional design as social network impact (and caregiver burden) may change over time. Further, treatment data was not collected due to the lack of ability to verify interventions, timing of interventions, and the lack of ability to adjust for regional therapeutic offering differences. The authors believe that successful treatment of DSRD would improve QOL and this represents a future area of study for the team*.”  and  “*Additionally, although the study achieved strong participation, it lacked longitudinal follow-up, precluding assessment of causality or temporal dynamics between caregiving burden and social network deterioration or improvement.*” |
| Participants were recruited from a neurology clinic and a DSRD Facebook support group. While this strategy ensured access to a relatively large sample, it introduces potential bias. Caregivers active in online support groups may represent those who are more socially engaged or more distressed, skewing the results. Similarly, caregivers recruited from clinical settings may differ in socioeconomic status, healthcare access, or severity of the child’s condition compared to those not seeking specialized care. This recruitment strategy may therefore limit the generalizability of findings to the broader population of caregivers. | Thank you for this comment. The authors are aware of this limitation, which is inherent to many survey-based studies in rare diseases.  This is noted in the limitations section as follows:  “*This study has several limitations which must be considered when interpreting the data. First, reliance on caregiver self-report introduces the possibility of recall bias, social desirability bias and limits diagnostic verification, particularly in the DSN group*.”  and  *“Second, the DSRD cohort included participants recruited from a disease-specific online support group, which may overrepresent caregivers experiencing higher distress or social withdrawal, reducing generalizability*.”  and  *“Demographic factors such as socioeconomic status, education, and geographic location were not uniformly collected, further limiting generalizability.”* |
| The study relied exclusively on caregiver self-reports through the DSRD Caregiver Distress Survey (CDS). Self-reported data are inherently vulnerable to recall bias, social desirability bias, and subjective interpretation of questions. For example, caregivers may underreport positive aspects of their social networks or overemphasize negative experiences due to heightened emotional distress. Without triangulation from objective measures—such as social network mapping, behavioral observations, or longitudinal tracking—the findings remain dependent on subjective perceptions. | Thank you for this comment. The authors are in agreement with the reviewer that self-reported survey-based data such as that presented in this study is inherently at risk for recall bias and a variety of other biases. The authors have noted this in the limitations section as follows:  “*This study has several limitations which must be considered when interpreting the data. First, reliance on caregiver self-report introduces the possibility of recall bias, social desirability bias and limits diagnostic verification, particularly in the DSN group*.” |
| Although the CDS included four open-ended questions, the scope was relatively narrow, focusing on adult friendships, social networks, spouse/partner impact, and social network size. Important dimensions of caregiver social functioning—such as workplace relationships, extended family dynamics, or participation in religious or cultural communities—were not explicitly explored. This omission may have overlooked significant areas of social support or strain that contribute to caregiver well-being. | Thank you for this comment. The author is correct in the narrower scope of the fields assessed. While open responses could have been subdivided into multiple coding schemas, the limited number of respondents in this study would have limited the ability to evaluate differences between groups statistically. This design may have limited the ability to detect other social support/strains that could contribute to caregiver well-being. As this study was exploratory in nature, and further follow up studies are already underway, we are hopeful to identify more nuanced themes in future studies.  This has been clarified in the limitations section as follows:  ‘*In addition, while the thematic coding process was consensus-based and methodologically rigorous, qualitative analysis remains inherently interpretive and may not fully capture the complexity of caregiver experiences. Further, the narrower nature of the coding may have oversimplified themes or omitted other areas of social support or strain*.” |
| Moreover, the thematic coding process, while systematic, involved reducing rich qualitative narratives into frequency counts. This conversion risks oversimplifying the complexity of caregiver experiences. Nuanced differences in the depth, intensity, or context of social withdrawal may have been lost in translation. For example, the meaning of “loss of community participation” may vary widely across cultural or geographic contexts, but such distinctions were not captured in the quantitative analysis. | Thank you for this comment. Similar to the authors’ response above, the study design may omit social themes that could impact caregivers in both positive and negative ways.  The authors have addressed this in the limitations section as follows:  ‘*In addition, while the thematic coding process was consensus-based and methodologically rigorous, qualitative analysis remains inherently interpretive and may not fully capture the complexity of caregiver experiences. Further, the narrower nature of the coding may have oversimplified themes or omitted other areas of social support or strain*.”  In addition, cultural and geographic interpretation of certain phrases and terms may limit response generation. This has been addressed in the limitation section as well with the following statement:  “*Further, the narrower nature of the coding may have oversimplified themes or omitted other areas of social support or strain. In addition, some language such as the use of the term “permanent” or “loss of community participation” can be hard to interpret both for those completing the survey and the researchers coding the data*.”  To aid in the interpretation of this in the results sections, the authors have condensed the sub-headings to just “*Qualitative CDS Item Responses”* and “*Qualitative Themes from Open Responses”.* |
| The study did not provide detailed demographic breakdowns of participants, such as age, gender, ethnicity, socioeconomic status, or geographic location. These factors are critical in shaping both caregiving experiences and social networks. Without accounting for diversity, the findings may disproportionately reflect the experiences of certain groups while neglecting others. For instance, rural caregivers may face different challenges than urban caregivers, and cultural norms may influence the extent to which social withdrawal is perceived or reported. | Thank you for identifying this clarity issue. The authors have added this information to Table 2 in a separate section. Unfortunately, information regarding geographic location and socioeconomic status was not collected for this study but does represent an area of future research.  The authors have noted this specifically in the limitations section of the discussion as follows:  *“Demographic factors such as socioeconomic status, caregiver education level, family structure and geographic location were not uniformly collected, further limiting generalizability.”*  The authors agree with the reviewer that this information would be helpful to understand if socioeconomic status (in particular) impacts stress level of caregivers who would be presumed to have to handle more of the direct care needs directly instead of outsourcing them.  This has also been expanded in the discussions section as follows:  “*The authors feel strongly that certain factors such as socioeconomic status may be important drivers of social network loss to explore in the future as discrepancies with primary caregivers not having financial resources to obtain additional care support*.” |
| Although the survey included questions on spouse/partner relationships, the findings were not statistically significant. This may reflect limitations in sample size, question framing, or analytic sensitivity rather than a true absence of impact. Given the extensive literature documenting marital strain in caregiving contexts, the lack of significant findings here suggests that the study may not have adequately captured the complexity of spousal dynamics. | Thank you for this comment. The reviewer is quite correct in noting that this is commonly observed in other studies. The authors have noted this in the discussion as follows:  “*While not statistically significant, the impact on spousal and partner relationships trended toward significance, indicating that the burdens of caregiving for a loved one with DSRD may also strain the most intimate of social ties*.”  and  “*Many of these concerns and themes were shared amongst the DSRD caregivers. Overall, the existing literature supports lower marital satisfaction, diminished cohesion, and in some cases, elevated risk of compared with parents of typically developing children*.”  Unique to this data set is that the strain on spouse/partner relationships is being compared to children with other neurological conditions and not otherwise healthy individuals with Down syndrome or typically developing children. As such, it is possible that the strain on the relationship inherent to caring for a child with Down syndrome and a neurological issue is not in excess of an individual with DSRD. As this can be interpreted bidirectionally, the authors have opted to simply present the data without inferring causality one way or another. |
| In addition, the study focused primarily on documenting negative impacts without exploring potential coping strategies, resilience factors, or interventions that might mitigate social isolation. Some caregivers may develop adaptive mechanisms or find strength in shared caregiving experiences, but these positive dimensions were not systematically analyzed. As a result, the study presents a predominantly deficit-based perspective. | Thank you for this comment. The reviewer is correct in noting that the predominant evaluation here is for negative impacts. Much of the original survey was based on report from families, parents, and caregivers in the clinical setting where individuals were frequently reporting negative sequelae associated with the care of individuals with DSRD. That said, the structure of the survey could also indicate that individuals did not have negative impacts (neutral). That said, this was very rarely reported in this cohort. |
| Tables. No acronyms in title and clarify always space and temporal location of this study, e.g., Children's Hospital Los Angeles and so on …. | Thank you for this comment. The authors have removed the acronyms from the titles of tables as suggested. The study was survey-based and thus not physically conducted at a center (just analysis). The authors have clarified where participants were recruited from in the methods as follows:  “*In the DSRD cohort, 141 (61.8%) were recruited from clinics and 87 (38.2%) were recruited from online groups compared to 94 (68.6%) and 43 (31.4%) in the DSN cohort, respectively (p=0.19).”* |
| The paper has a lot of tables that are difficult to digest, some of them can be put in appendix and inserting in the text the most important ones to improve the readability… | Thank you for this comment. The authors believe all information presented in tables is critical to understanding the data collected. The authors have simplified the tables for readability and also reduced the number of decimal points as suggested by other reviewers. The reformatted tables are now significantly more easy to read. |
| To reiterate, avoiding a lot of sub-headings that create fragmentation of the paper. | Thank you for this comment. The authors have removed nearly all subheadings in the manuscript aside from the requested breakdowns in the methods section by this reviewer. |
| *Discussion* | |
| First, authors have to synthesize the main results in a simple table to be clear for readers and then show what this study adds compared to other studies. Although the Results section provides a detailed description of the data collected and analyzed, there needs to be a more critical synthesis and comparison of the findings with the literature. | Thank you for this comment and identifying this clarity issue. The authors have revised the first paragraph of the discussion to better synthesize the results as suggested. The authors have revised the first paragraph significantly and this now reads as follows:  *“In this cross-sectional comparison of caregivers of individuals with DSRD versus DSN, we found consistent evidence of social network strain across multiple domains, with the clearest between-group differences involving social withdrawal, reduced community participation, and perceived enduring loss of ties. Prior caregiver studies in neurodevelopmental and neurologic conditions describe isolation, stigma, and reduced participation; our contribution is quantifying these social-network impacts in DSRD relative to a DSN comparison group, suggesting a distinct pattern of community disengagement and perceived longer-lasting network contraction. Caregivers described caregiving demands as associated with reduced social engagement and fewer opportunities to sustain friendships and community participation, which, when repeatedly reinforced, were perceived as progressive network loss. Together, these findings suggest a coherent pattern in which caregiving constraints, social withdrawal, and reduced community participation co-occur with perceptions of sustained loss of social ties, while acknowledging that longitudinal data are needed to assess persistence over time. Clinically, these results support routine screening for caregiver isolation and proactive linkage to respite, peer support, and navigation resources; approaches that may help preserve social connectedness early in the DSRD course.”* |
| The discussion section has to interpret and describe the significance of your findings in relation to what was already known about the research problem being investigated and explain any new understanding or insights that emerged from your research. The discussion has better to connect to the introduction through the research questions and the literature you reviewed. | Thank you for this comment and identifying this clarity issue. The authors have expanded the discussion to better explain this topic. Expanded areas now read as follows:  *“More broadly, this suggests that the challenges associated with caregiving lead to significant and lasting consequences that permanently alter the caregiver’s social landscape and, potentially, mental health. These data have never been reported in individuals with DSRD and a question that has emerged is what the longitudinal impact of these findings is based on clinician-based diagnosis and treatment. Future studies evaluating this effect could inform the need for early identification of caregiver social impact and potential intervention.”*  and  *“Higher levels of stress are associated with the care of children and adults with DS. Social network size reductions were more pronounced in the DSRD cohort, primarily driven by a time and energy constraints and reduced invitations from existing social networks. It is the author’s hypothesis that this pattern is a negative feedback loop wherein higher levels of care for loved ones with DSRD leads to decreased time and ability to engage socially which subsequently leads to decreased invitations for engagement from the existing social network. This pattern of decreased time to engage in social activity has been previously reported in individuals with DS and the overall negative feedback loop of social isolated is established in caregivers of those with chronic neuropsychiatric disease. It is presumed that this phenomenon is not attributable to the individual with DS as social networks have been previously reported to be static over time in this population.”* |
| The discussion clearly has to explain how this study advances these research topics and previous study by some of these authors. | Thank you for identifying this clarity issue. The authors have expanded this in the discussion in several locations as follows:  *“The results of this study demonstrate how the unique demands of caregiving directly and indirectly lead to a decline in social ties, community engagement, and, ultimately, a lasting loss of social networks. This research provides a framework for understanding the trajectory from caregiving constraints to social network degradation and ultimately, the permanent loss of social connections. In addition, it provides a basis for early identification and potential intervention^3, 4^.”*  and  “*This diminished capacity for social engagement is not merely a temporary inconvenience but is associated with a permanent loss of social ties. More broadly, this suggests that the challenges associated with caregiving lead to significant and lasting consequences that permanently alter the caregiver’s social landscape and, potentially, mental health.”*  and  *“The differentiation between caregiving for a child with DSN versus DSRD is a critical distinction. While similar themes of social isolation, decreased community participation, and relationship strain are reported by caregivers of children with other neurological conditions, the findings of this study suggest that the unique and often severe challenges of DSRD caregiving impose more profound constraints on families, particularly in terms of social engagement and involvement in the community.”*  The authors have also changed the verbiage of the conclusions section to make these impacts and implications clearer. Please see response below for additional details regarding this. |
| Comment on whether the results were expected for each set of findings; go into greater depth to explain unexpected findings. Moreover, either compare your results with the findings from other studies or use the studies to support your results. | Thank you for this comment. The findings in DSRD are novel as this has not been previously reported on in the literature. The authors have expanded on this in the 2^nd^ paragraph of the discussion as follows:  “*The most statistically significant findings in this study centered on the erosion of social ties. Specifically, caregivers of individuals with DSRD reported increased social withdrawal and isolation and a loss of community participation and support, suggesting a disengagement from social functions and interactions within their communities. These challenges align with a broader finding that caregiving constraints, particularly a lack of time and energy, affect a caregiver’s ability to maintain their social networks. This diminished capacity for social engagement is not merely a temporary inconvenience but is associated with a permanent loss of social ties. More broadly, this suggests that the challenges associated with caregiving lead to significant and lasting consequences that permanently alter the caregiver’s social landscape and, potentially, mental health. These data have never been reported in individuals with DSRD and a question that has emerged is what the longitudinal impact of these findings is based on clinician-based diagnosis and treatment. Future studies evaluating this effect could inform the need for early identification of caregiver social impact and potential intervention.”*  In addition, the authors have expanded on direct comparisons to other neurological conditions in the third paragraph of the discussion as follows:  *“The differentiation between caregiving for a child with DSN versus DSRD is a critical distinction. While similar themes of social isolation, decreased community participation, and relationship strain are reported by caregivers of children with other neurological conditions, the findings of this study suggest that the unique and often severe challenges of DSRD caregiving impose more profound constraints on families, particularly in terms of social engagement and involvement in the community. For instance, studies on children with cerebral palsy show significantly lower community and home participation, with caregivers identifying physical, cognitive, and social barriers. These limitations include a lack of transportation, services, and financial support as key factors. Likewise, research on caregivers in the broader DSN population, such as mothers of children with various long-term care needs, highlights similar challenges such as social isolation and distance from friends stemming from a lack of knowledgeable support networks and gaps in respite services^15^. However, while these studies describe significant barriers to social engagement, they do not typically emphasize the permanence of the social loss. This distinction is crucial because although caregivers across many conditions in the DSN group face obstacles to community participation, the data from our DSRD cohort suggests their social losses are not just situational but represent a lasting deterioration of their social networks. This degree of permanent social loss may indicate a more severe trajectory for DSRD caregivers. The severe impacts of DSRD are not only limited to caregiving tasks, but also cascade into the caregiver’s social, relational, and emotional life. Thus, interventions that support the individual’s medical needs while also actively protecting the caregiver's social and mental health are urgently needed.”*  and  *“Higher levels of stress are associated with the care of children and adults with DS. Social network size reductions were more pronounced in the DSRD cohort, primarily driven by a time and energy constraints and reduced invitations from existing social networks. It is the author’s hypothesis that this pattern is a negative feedback loop wherein higher levels of care for loved ones with DSRD leads to decreased time and ability to engage socially which subsequently leads to decreased invitations for engagement from the existing social network. This pattern of decreased time to engage in social activity has been previously reported in individuals with DS and the overall negative feedback loop of social isolated is established in caregivers of those with chronic neuropsychiatric disease. It is presumed that this phenomenon is not attributable to the individual with DS as social networks have been previously reported to be static over time in this population.”* |
| A claim for how the results can be applied more generally in other US contexts and also in other nations. | Thank you for this comment. The authors have added this information to the conclusions section of the manuscript under the heading of “Health Policy Implications Based on Incentive Systems for Caregiver Social Networks”. A challenge with this study is that it did not study application and thus the discussion on this must be limited to a “next step” which is integrated in with a “future research” section as proposed by this reviewer. This section now reads as follows:  *“Health Policy Implications Based on Incentive Systems for Caregiver Social Networks: These data support treating caregiver connectedness as a health-relevant resource that systems can protect or erode. Incentive-aligned policies could include 1) health system protocols (screening for caregiver isolation; referral to respite, navigation, and mental health supports, 2) clinic-embedded peer supports); payer coverage for care coordination, respite, and 3) structured caregiver programs, and employer/community supports (flexible scheduling, protected leave, accessible respite).*  *Future Research: Logical next steps are longitudinal, multi-site studies with stronger diagnostic characterization, detailed caregiver context, validated social-network measures plus interviews, and analyses of moderators (treatment course, clinical response, respite access). Longitudinal study design will also afford the ability to track impacts over treatment periods which may yield unique fluctuations and less “permanence” of findings as reported in this study. Further, caregiver intervention studies can test whether incentive-aligned support can prevent network loss and downstream caregiver distress.”*  Of note, addressing national v international suggestions is beyond the scope of this manuscript given the heterogeneity of health systems across the world although is certainly a topic the authorship group will explore in future studies. |
| Describe lessons learned, proposing recommendations based on incentive systems in Caregiver Social Networks that can help improve the problem and topic under study, highlighting best practices of health and social policies. | Thank you for this comment. The authors did not assess incentive systems in this study and thus there would be limited assessment of the potential for this based on the data provided. That said, the authors have discussed this topic as a next steps section in the conclusions as listed below. |
| Conclusion so short is useless. Conclusion has not to be a summary, but authors have to focus on manifold limitations of this study (moving here the lines 278-293) and not in previous section. The Conclusion does not adequately discuss the theoretical and health policy implications of the study. Discuss how the gap in the literature has been addressed about the research problem, compared to similar studies. Make sure you create 3 subsections in the Conclusion: 1) Theoretical Implications, 2) Health Policy Implications based on incentive systems for Caregiver Social Networks, and 3) Future Research. | Thank you for this comment. The authors have revised the conclusions to the sub-headings that the reviewer requested. The revised conclusion now reads as follows:  *“Theoretical Implications*  *This study advances the DSRD caregiving literature by focusing on caregiver social networks (not only global burden). Across domains (friendships, partner relationships, network size), caregivers described network contraction driven by caregiving intensity, social withdrawal, and reduced participation—often experienced as lasting. By comparing DSRD with a DSN group using a shared framework, we address a key gap: how DSRD-specific caregiving demands shape social connectedness compared with other neurologic conditions in Down syndrome.*  *Health Policy Implications Based on Incentive Systems for Caregiver Social Networks: These data support treating caregiver connectedness as a health-relevant resource that systems can protect or erode. Incentive-aligned policies could include 1) health system protocols (screening for caregiver isolation; referral to respite, navigation, and mental health supports, 2) clinic-embedded peer supports); payer coverage for care coordination, respite, and 3) structured caregiver programs, and employer/community supports (flexible scheduling, protected leave, accessible respite).*  *Future Research: Logical next steps are longitudinal, multi-site studies with stronger diagnostic characterization, detailed caregiver context, validated social-network measures plus interviews, and analyses of moderators (treatment course, clinical response, respite access). Longitudinal study design will also afford the ability to track impacts over treatment periods which may yield unique fluctuations and less “permanence” of findings as reported in this study. Further, caregiver intervention studies can test whether incentive-aligned support can prevent network loss and downstream caregiver distress.”* |
| Overall, then, the paper is interesting, but the structure is confusing. Theoretical framework is weak, and some results create confusion… structure of the paper has to be improved; study design, discussion and presentation of results have to be clarified using suggested comments. | Thank you for this comment. As noted above, the authors have spent significant time in reducing sub-headings, clarifying framework, and improving clarity on topics presented. |
| I kindly recommend revising the paper by thoroughly addressing all comments provided, including engaging with the suggested readings. These references should be carefully reviewed and appropriately incorporated into both the main text and the bibliography. I will conduct a detailed evaluation of the revised version, and only then will the paper be considered for further review. | Thank you for this comment. The authors have added the references suggested by the reviewer which were very beneficial for improving the background and flow of the report. |

Reviewer #3: I have to start with thanking the authors for making me aware of the issue at hand, i.e. the (possible) differences between DSRD and 'regular' DS. The authors have made a commendable effort to study the impact for the caregivers, but unfortunately, I do not think the manuscript in its current form meets the criteria for publication.

| **Reviewer Comment** | **Author Responses** |
| --- | --- |
| The study in this form confuses the assessment of the patients and the viewpoints of the caregivers to a degree that makes the results hard to interpret and understand. This firstly stems from the use of a qualitative measure, and therefore qualitative results that had to be coded into quantitative data in order to be analysed quantitatively. Ideally, this step would have been unnecessary by simply using a quantitative measure. In principal, I think researchers should use quantitative measures if they want to perform quantitative analyses, and use qualitative measures, if they want to establish insight, rather than analysis. But that point has passed and I can therefore look past it. | Thank you for this comment. The reviewer is quite correct in noting that a preferred assessment would be to use qualitative measures explicitly. This data set emerged from a quantitative assessment previously published that had qualitative fields for analysis. While this was not part of the original plan for analysis, the responses there were quite striking and thus this study represents a follow up from this data set. The authors have planned a follow up study (based off of this data) that is strictly qualitative in nature. |
| The characteristics of the caregivers, who are the actual research subjects, are not presented or discussed. In order for the reader to understand and interpret the results, they would need to know about the demographics (e.g.: the DSRD patients are significantly older: does that translate into older caregivers?), the family setup (e.g.: how many caregivers are present in the family? How many (older) siblings?) etc. I feel this is a major shortcoming and should be corrected: treat the caregivers as your sample and present their relevant characteristics. | Thank you for identifying this clarity issue. The reviewer is quite correct in noting that this original data set focused heavily on the individuals with neurological conditions demographics as opposed to the caregivers themselves. The authors have added caregiver demographics into Table 2. Notably, while age is obviously different, the general racial and ethnic composite of the group is similar to the individuals with DSRD and neurological conditions  Unfortunately, data on socioeconomic status, and family arrangements were not available in this study as it was exploratory. This has been acknowledged in the limitations section of the discussion and has been included as fields for our follow-up studies.  “*Demographic factors such as socioeconomic status, caregiver education level, family structure and geographic location were not uniformly collected, further limiting generalizability*.” |
| It is then confusing that the results are split into 'Responses of CDS items' and 'Qualitative responses'. | Thank you for identifying this clarity issue. We now first present “Quantitative CDS Item Responses,” which describes the closed-ended caregiver burden and social network items, followed by “Qualitative Themes from Open-ended Questions,” which describes the coding results and representative quotations with a new section added corresponding to the latter. We removed redundant subheadings and ensured that each section clearly signals whether it refers to closed-ended vs open-ended data. |
| A coding process introduces so much fuzziness, that I don't think double decimals are in order. | Thank you for this comment. We are hopeful that the clarifications on the coding process are beneficial to the reviewer. A single decimal point is offered for percentages and we have amended this to *p* values as well in tables as well. The only exception to this is where <0.001 exists. |
| At the end of the introduction, I don't feel the point that DSRD symptoms are unique compared to symptoms of other neurological diagnoses had been made sufficiently | Thank you for identifying this clarity issue. The authors have clarified the unique phenotype differences between individuals with DSRD and DSN as follows:  *“Previous research has revealed that* *parents of children with neurological conditions such as epilepsy, cerebral palsy, and autism spectrum disorder (ASD), experience impacts to their social networks as well. These conditions are associated with seizure, spasticity, and neurocognitive issues which are different than the largely neuropsychiatric symptoms reported in individuals with DSRD.”* |
| The coding process was not sufficiently described for me. I would like more info about how the number of codes was established and how many disagreements there were between the two coders. | Thank you for identifying this clarity issue. The authors have expanded the methodology of coding as follows:  “*To analyze these qualitative responses, a thematic coding process was utilized.* *We analyzed caregiver free-text responses to four open-ended survey items addressing impacts of the individual’s condition on adult friendships, marital/partner relationships, perceived social support, and perceived changes in social network size. Caregivers were the unit of analysis. Responses were exported verbatim, de-identified, and segmented into “meaning units” (a sentence or clause expressing a single idea); a single response could contribute multiple meaning units. We conducted a semantic thematic analysis using a hybrid inductive–deductive coding strategy: an initial deductive code “start list” was derived from the survey domains (e.g., friendship disruption, partner strain, social support changes, network contraction), and inductive codes were added iteratively to capture unanticipated content (e.g., stigma-related withdrawal, time burden, role overload, health system navigation). Two coders independently reviewed and coded an initial subset of responses (15%) to develop a shared codebook specifying code definitions, inclusion/exclusion criteria, and exemplar quotations. The codebook was refined through iterative consensus meetings with a group of four authors until stable, after which the full dataset was coded with multiple codes permitted per meaning unit when appropriate. Discrepancies between coders were resolved by consensus discussion; if consensus could not be reached, the senior author adjudicated. Following coding, related codes were collated into higher-order themes through iterative review, with themes defined (scope and boundaries), checked against the full dataset, and refined to ensure internal coherence and distinctiveness across themes. To aid interpretability, the authors reported each theme with representative quotations; where frequencies are provided, they reflect the proportion of caregiver responses mentioning a code/theme and are intended as descriptive summaries rather than prevalence estimates. Finalized coded themes are as follows*:   1. *Quality of Adult Friendships: a) Social Withdrawal and Isolation; b) Emotional Fatigue of Caregivers; c) Social Disconnection and Exclusion; d) Negative Mental Health Impact; e) Difficulty Leaving Home or Including Loved One with DSRD* 2. *Impact on Social Networks: a) Social Withdrawal and Isolation; b) Caregiving Constraints; c) Behavioral Barriers & Stigma; d) Loss of Community Participation and/or Support; e) Diminished Size of Social Network* 3. *Spouse/Partner Impact: a) Emotional and Interpersonal Strain; b) Disagreements on Caregiving & Treatment; c) Lack of Time and Intimacy; d) Caregiving Stress-Related Relationship Deterioration; e) Unequal Roles or Perceived Imbalance* 4. *Social Network Size: a) Time and Energy Constraints; b) Friends' Discomfort or Inability to Relate; c) Reduced Invitations from Social Network; d) Shift to Condition-Specific Support Network; d) Permanent Loss of Social Ties*   *All open-ended responses were coded using these themes, and the frequency of each code was tallied for both the DSRD and DSN groups. Where reported, code frequencies reflect the proportion of responses in which a theme was mentioned and are presented to aid comparison between cohorts rather than as population prevalence estimates*.” |
| This point may potentially be more important, but I can only interpret that after I know the caregiver characteristics: what would the authors make of the fact that the age difference between DSRD and DSN patients is almost one SD? Could this fact alone explain some of the differences? | Thank you for this comment. There were no differences in caregiver characteristics that were collected, or which caregiver was reporting the data. This is now more clearly reported in Table 2 and includes *p* values.  The differences in ages of the cohort are more related to the conditions evaluated as DSRD is frequently diagnosed in the late teens and other neurological conditions surveyed present earlier in the time course. The most important feature that allows for comparison between the two groups is the duration of the disease process noting that DSRD continues to have major impacts on social networks after diagnosis whereas other neurological conditions may not. This was not statistically different. The authors do not feel that this influenced the caregiver responses as the *p* values reported are adjusted analyses.  The authors have also explicitly noted that the analysis do take in to account age:  *“A p-value of less than 0.05 was considered to be statistically significant, with values trending toward this threshold noted for further consideration. All p-values are reflected of adjusted analysis to control for age, ethnicity and race.”* |
