# Supplementary material for: Caregiver-reported social impacts in down syndrome regression disorder
Source: PLoS One. 2026 Feb 4;21(2):e0342148. doi: 10.1371/journal.pone.0342148 (PMC12871964; doi:10.1371/journal.pone.0342148)
Supplement: Attachment — Submitted filename: Author Responses R2.docx [file pone.0342148.s005.docx]

January 9^th^, 2026

Dear Editors of *PLoS One* and Dr. Roggen,

Thank you for the opportunity to revise our manuscript entitled *“Impact of Down Syndrome Regression Disorder on Caregiver Social Networks: A Cross-Sectional Study at a Tertiary Pediatric Center in the United States”.* The authors sincerely appreciate the time spent in review of this manuscript and have responded in a point-by-point manner to the feedback provided below. The authors believe that the feedback provided, particularly by the editor, significantly improve the quality of the manuscript and we hope that the revised version is acceptable for publication.

Most Sincerely,

Jonathan D. Santoro, MD

Editor’s Comments: In particular, the current manuscript frequently presents subjective, cross-sectional, self-reported perceptions as if they reflect objective and structural properties of caregiver social networks. The study does not directly measure social network structure, size, density, or dynamics, but rather caregiver perceptions and narratives regarding social experiences.

| **Editor Comment** | **Authors Response** |
| --- | --- |
| The manuscript should therefore consistently frame findings as perceived or self-reported social impacts, and avoid language implying objective, causal, or structural network change. Related to this, the repeated use of the term “permanent” to describe social loss is not supported by the cross-sectional design and should be revised or softened. Claims regarding permanence, progression, or enduring network contraction must be clearly presented as caregiver perceptions rather than empirically demonstrated longitudinal outcomes. | Thank you for this comment. The authors sincerely appreciate this feedback and agree with this need for more clear distinction. The authors have made the following changes to the manuscript to address the editors comments:   1. The title of the manuscript has been changed to highlight that the data presented is self-reported, not objectively obtained. The title now reads “*Caregiver-Reported Social Impacts in Down Syndrome Regression Disorder*” 2. In the abstract, the authors have clarified that prior studies have also focused on subjective report of negative impacts. The 2^nd^ sentence of the background section of the abstract now reads as follows: *“DSRD increases demands on caregivers, leading to sleep disturbances, financial distress, and negative impacts on caregiver-reported social connections and perceived social support*. *The goal of this study was to characterize the caregiver-reported impacts of DSRD on social networks by comparing their experiences to those of caregivers of individuals with DS and other neurological disorders (DSN).”* 3. In the methods section of the abstract, the authors have also specified the self-perception component of the fields studied. This section now reads as follows: “*Participants completed the DSRD Caregiver Distress Survey (CDS), which included four qualitative, open-ended questions focused on self-perception of adult friendships, social network impact, spouse/partner impact, and social network size*.”   Further, this was also specifically stated in the abstract methods as follows: “*Responses were analyzed using thematic coding; resulting theme frequencies summarize caregiver-reported perceptions and narratives and do not represent objectively measured social network structure.”*   4. In the abstract, the permanent statement in the results has been replaced and softened as requested. This sentence now reads as follows: “*Caregivers in the DSRD group were significantly more likely to report “Social Withdrawal and Isolation” (43.2% vs. 17.9%, p=0.006), “Loss of Community Participation and/or Support” (16.7% vs 4.5%, p=0.043) and a "Perceived Enduring Loss of Social Ties" (35.3% vs. 8.7%, p=0.002) compared to the DSN group*.”   Of note, the authors have also used this terminology throughout the manuscript, figures, and tables in multiple locations.   5. In the abstract’s conclusion, the verbiage of permanence and marked impact has also been softened. The first sentence now reads as follows: “*This study's findings reveal a significant and complex process of perceived social disengagement among caregivers describing social withdrawal and loss of social ties that they experienced as enduring*.” 6. In the introduction, the authors have made clearer that the purpose of this study was characterization of self-reported social impacts. This sentence now reads as follows:   *“The purpose of this study was to* *characterize caregiver-reported social impacts associated with caring for an individual with DSRD and compare these reported impacts with a heterogeneous caregiver group of individuals with DS and other neurological disorders (DSN) [12]. This study also characterized caregiver perceptions regarding changes in the quality of adult friendships, impact on social networks, impact on spouse/partner, and social network size*.  This has also been clarified in the introduction further as follows: “*Understanding how the unique demands of DSRD caregiving may be associated with caregiver perceptions of reduced social support, is important in highlighting the overall care burden and mental health outcomes.”*   7. To allow for persistent use of caregiver-reported results, the authors have also made this edit in the results section: “*Caregiver-reported results indicated a significantly higher percentage of DSRD caregivers reported a 'Perceived Enduring Loss of Social Ties' (35.3% vs. 8.7%, p=0.01, [0.1, 0.4]) compared to the DSN group (****Figure 2b****).”* 8. The authors have also revised the term “objective responses” in reference to Table 4 as follows in the results section: “*The DSRD and DSN self-reported responses are summarized in* ***Table 4***.” 9. In the first paragraph of the discussion, the introductory sentence has been tempered to read as follows: “*In this cross-sectional comparison of caregivers of individuals with DSRD versus DSN, we found consistent caregiver-reported perceptions of social strain and reduced social connectedness across multiple domains, with the clearest between-group differences involving social withdrawal, reduced community participation, and perceived enduring loss of ties*.” 10. In the discussion, another highlighting of self-report was made with regards to network size. This sentence now reads as follows: “*This distinction is crucial because although caregivers across many conditions in the DSN group face obstacles to community participation, DSRD caregivers more frequently reported social losses that they experienced as enduring; however, persistence and temporality cannot be determined from this cross-sectional survey. This degree of perceived enduring social loss may reflect higher caregiving burden among DSRD caregivers in this respondent sample.”* 11. In the third sentence, the authors have also clarified the self-perception aspect of this and added in increased distinction between the DSRD and DSN cohorts (point two by the editor): *“Prior caregiver studies in neurodevelopmental and neurologic conditions describe isolation, stigma, and reduced participation; our contribution is describing caregiver-reported social impacts in the DSRD cohort relative to a heterogeneous DSN comparison group. While between-group differences were observed in reported community participation and reported loss of social ties, these findings should be interpreted cautiously given the self-reported nature of outcomes and the heterogeneity of neurologic diagnoses within the DSN comparator, which limits disorder-specific inference [12]. While these differences are statistically significant within this sample, they represent subjective narratives and should not be used to infer structural network differences between disorders.”* 12. Further, the authors have softened the findings reported in the 2^nd^ paragraph of the discussion and added that losses were not permanent but perceived as enduring as follows: “*The most statistically significant findings in this study centered on caregivers’ descriptions of loss of social ties that they experienced as enduring*.”   Further in this paragraph: “*This diminished capacity for social engagement is not merely a temporary inconvenience but was described by caregivers as contributing to an enduring loss of social ties*.”  and, removed the concept of permanence from this sentence:  *“More broadly, these results suggest that the challenges associated with caregiving lead to significant consequences that may alter the caregiver’s social experiences and perceived support.”*  and  *“This distinction is crucial because although caregivers across many conditions in the DSN group face obstacles to community participation, DSRD caregivers more frequently reported social losses that they experienced as enduring; however, persistence and temporality cannot be determined from this cross-sectional survey. This degree of perceived enduring social loss may reflect higher caregiving burden among DSRD caregivers in this respondent sample.”*  and  “*We cannot attribute perceived caregiver social changes to any single factor in this cross-sectional design as prior work on social networks being “static” in people with DS may not generalize to caregiver networks [23]”*   13. In the limitations section, the authors have added an explicit statement about lack of social network size measurements. This statement reads as follows: “*Importantly, we did not directly measure social network structure (size, density, or dynamics) using formal social network analysis; findings therefore reflect caregiver perceptions and narratives rather than objectively observed network properties or causal change*.” 14. In the conclusions, the authors have made more straightforward statements regarding the reductions as follows: “*Across domains (friendships, partner relationships, network size), caregivers described perceived reductions in social connectedness and participation.”*   and  *“Longitudinal study design will also afford the ability to track impacts over treatment periods which may yield unique fluctuations and assess if self-reported perceptions of loss are as enduring as reported in this study.”* |
| Finally, interpretations comparing the DSRD and DSN groups should be further tempered to reflect the heterogeneity and self-reported nature of the DSN comparator, and the limitations of causal or disorder-specific inference. | Thank you for this comment. The discussion has been edited in a number of locations to provide more softened interpretations and highlight the limitations of causal and/or disorder specific inference. The following edits have been made to the discussion:  *“Prior caregiver studies in neurodevelopmental and neurologic conditions describe isolation, stigma, and reduced participation; our contribution is describing caregiver-reported social impacts in the DSRD cohort relative to a heterogeneous DSN comparison group. While between-group differences were observed in reported community participation and reported loss of social ties, these findings should be interpreted cautiously given the self-reported nature of outcomes and the heterogeneity of neurologic diagnoses within the DSN comparator, which limits disorder-specific inference [12].”*  and, to lead off the 3^rd^ paragraph:  “*Comparisons between caregivers of individuals with DSRD and those in the DSN group should be interpreted cautiously*.”  and in the same paragraph:  *“While similar themes of social isolation, decreased community participation, and relationship strain are reported by caregivers of children with other neurological disorders, in this respondent sample, caregivers in the DSRD group more frequently perceived constraints on social engagement than caregivers in the DSN group.”*  In the limitations sections, the authors have also expanded our first point to read as follows: “*First, reliance on caregiver self-report introduces the possibility of recall bias and social desirability bias and limits diagnostic verification, particularly in the DSN group. Because the DSN comparator is heterogeneous and diagnoses/outcomes were not clinically adjudicated within this study, between-group differences should be interpreted as differences in caregiver-reported experiences among respondents rather than disorder-specific effects attributable uniquely to DSRD*.”  This has also been expanded further down to state: “*Additionally, the DSN comparison group was intentionally heterogeneous, encompassing various neurologic conditions; this limits direct comparability and constrains disorder-specific inference, as observed differences may reflect differences in diagnosis mix, comorbidity burden, severity, and caregiver context rather than DSRD itself*.”  and  “*Additionally, the cross-sectional design and lack of longitudinal follow-up preclude assessment of causality or temporal dynamics; accordingly, findings should be interpreted as associative caregiver reports rather than evidence of causal or progressive disorder-specific social network change*.”  Finally, the authors have changed the framework of the conclusions section (theoretical implications) as follows: “*By comparing caregiver reports in the DSRD cohort with those from a heterogeneous DSN group using a shared framework, we provide initial descriptive evidence of differential perceived social impacts; however, disorder-specific inference will require longitudinal studies with more diagnostically homogeneous comparators and clinically verified diagnoses*.” |

Reviewer #1: All comments have been addressed

Reviewer #2: No response

- As noted above at the request of the editor, the authors have taken efforts to improve readability and soften the statements made with regards to causal links and limitations.
