# Supplementary material for: Caregiver-reported social impacts in down syndrome regression disorder
Source: PLoS One. 2026 Feb 4;21(2):e0342148. doi: 10.1371/journal.pone.0342148 (PMC12871964; doi:10.1371/journal.pone.0342148)
Supplement: Attachment — Submitted filename: Author Responses R3.docx [file pone.0342148.s006.docx]

January 15^th^, 2026

Dear Editors of *PLoS One* and Dr. Roggen,

Thank you for the opportunity to revise our manuscript entitled *“Caregiver-Reported Social Impacts in Down Syndrome Regression Disorder”.* The authors sincerely appreciate the time spent in review of this manuscript and have responded in a point-by-point manner to the feedback provided below. The authors believe that the feedback provided, particularly by the editor, significantly improve the quality of the manuscript and we hope that the revised version is acceptable for publication.

Most Sincerely,

Jonathan D. Santoro, MD

Editor’s Comments: There remain some smaller issues that are not fully addressed:

| **Editor Comment** | **Authors Response** |
| --- | --- |
| Remove “ties” | Thank you for this comment. The authors have removed the term “ties” and replaced it with “connections” in multiple instances. This has also been reflected in associated tables and figures. |
| Remove “network size”, replace with “relationships,” “contacts,” “perceived shrinkage of social world” | Thank you for this comment. The authors have made the requested edits with the following changes reflected in the text:  Abstract:  *“The goal of this study was to characterize the caregiver-reported impacts of DSRD on social relationships by comparing their experiences to those of caregivers of individuals with DS and other neurological disorders (DSN).”*  *“Participants completed the DSRD Caregiver Distress Survey (CDS), which included four qualitative, open-ended questions focused on self-perception of adult friendships, social relationship impact, spouse/partner impact, and perceived social contacts size.”*  *“In the DSRD cohort, a high-level overview revealed that 65.66% of responses reported a negative impact on adult friendships, while 71.21% reported a negative impact on social relationships.”*  *“A negative impact on spouse/partner relationships was reported in 51.53% of responses, and a perceived shrinkage of social world was found in 52.82%.”*  Introduction:  *“This can cause caregivers to experience sleep disturbances, financial distress, and negative impacts to family dynamics and social relationships, among other challenges. The time-intensive nature of care demands means that individuals with DSRD require significantly more support than they did prior to their onset of symptoms, with potential cascading negative effects on caregivers and their social relationships. While previously published literature has identified caregiver burden, dedicated exploration of the impact on social relationships of caregivers is unknown.”*  *“Additionally, caregivers have reported increased social exclusion and stigma, resulting in a significant decrease of their social relationships by both contacts and quality”*  *“This study also characterized caregiver perceptions regarding changes in the quality of adult friendships, impact on social contacts, impact on spouse/partner, and social relationships”*  Methods:  *“The survey included a section called the DSRD Caregiver Distress Survey (CDS) which featured four specific open-ended qualitative questions designed to capture the nuanced impact of caregiving on a caregiver's social relationships”*  *“We analyzed caregiver free-text responses to four open-ended survey items addressing impacts of the individual’s condition on adult friendships, marital/partner relationships, perceived social support, and perceived changes in social relationships.”*  *“We conducted a semantic thematic analysis using a hybrid inductive–deductive coding strategy: an initial deductive code “start list” was derived from the survey domains (e.g., friendship disruption, partner strain, social support changes, social relationship and contact contraction), and inductive codes were added iteratively to capture unanticipated content (e.g., stigma-related withdrawal, time burden, role overload, health system navigation).”*  Results:  *“Notably, caregivers of individuals with DSRD were more likely to report perceived declines in the quality of adult friendships (64.4% vs. 29.2%, p < 0.001), overall social relationships (71.1% vs. 34.3%, p < 0.001), and marital relationships (52.0% vs. 19.7%, p < 0.001) compared with DSN caregivers.”*  *“Additionally, a greater proportion of DSRD caregivers reported a perceived shrinkage of their social world (54.1% vs. 38.0%, p = 0.01).”*  *“Out of 198 total responses regarding the Quality of Adult Friendships, 130 (65.6%) reported a negative impact. For Social Relationships Impacted, 141 out of 198 responses (71.2%) indicated a negative impact. Regarding Spouse/Partner Impact, 101 out of 196 responses (51.5%) were negatively impacted. Finally, for Perceived Shrinkage of Social World, 103 of 195 responses (52.8%) reported a negative impact. The DSRD and DSN self-reported responses are summarized in Table 4.”*  *“Qualitative responses regarding social relationships were provided by 100 DSRD participants and 44 DSN participants. Caregivers reported impacts on various aspects of their social interactions (Figure 2b).”*  *“Regarding perceived social contacts, 51 DSRD caregivers and 46 DSN caregivers provided written responses.”*  *“"Reduced Invitations from Social Contacts" was more prevalent in the DSRD group (19.6% vs. 8.7%, p=0.38, [-0.1, 0.2]).”*  Discussion:  *“Caregivers described caregiving demands as associated with reduced social engagement and fewer opportunities to sustain friendships and community participation, which, when repeatedly reinforced, were perceived as progressive contact/relationship loss.”*  *“These challenges align with a broader finding that caregiving constraints, particularly a lack of time and energy, affect a caregiver’s ability to maintain their social relationships.”*  *“Likewise, research on caregivers in the broader DSN population, such as mothers of children with various long-term care needs, highlights similar challenges including social isolation and distance from friends stemming from a lack of knowledgeable support systems and gaps in respite services”*  *“Caregivers more frequently reported a perceived shrinkage of the social world in the DSRD cohort, primarily driven by a time and energy constraints and reduced invitations from existing social relationships.”*  *“It is the author’s hypothesis that this pattern is a negative feedback loop wherein higher levels of care for loved ones with DSRD leads to decreased time and ability to engage socially which subsequently leads to decreased invitations for engagement from the existing social relationships.”*  *“We cannot attribute perceived caregiver social changes to any single factor in this cross-sectional design as prior work on social relationships being “static” in people with DS may not generalize to caregiver relationships [23].”*  *“In addition, this study is limited by its cross-sectional design as social impacts (and caregiver burden) may change over time.”*  *“The authors feel strongly that certain factors such as socioeconomic status may be important drivers of perceived shrinkage of the social world and warrant exploration in the future as discrepancies with primary caregivers not having financial resources to obtain additional care support.”*  *“Additionally, the cross-sectional design and lack of longitudinal follow-up preclude assessment of causality or temporal dynamics; accordingly, findings should be interpreted as associative caregiver reports rather than evidence of causal or progressive disorder-specific social relationship change.”*  Conclusions:  *“This study advances the DSRD caregiving literature by focusing on caregiver social relationships (not only global burden). Across domains (friendships, partner relationships, social contacts), caregivers described perceived reductions in social connectedness and participation”*  Table 1:   1. Column 2 title change to: *Impact on Social Relationships* 2. Decreased Social Contacts 3. Column 4 title change to: *Perceived Shrinkage of Social World* 4. *Reduced Invitations from Social Contacts* 5. *Shift to Condition-Specific Support Relationships*   Table 3:   1. Subcategory title changed to: *Social contacts and relationships.* 2. *Social relationships impacted* 3. *Perceived shrinkage of social world.* The authors also changed this to a yes/no response to match the verbiage change.   Table 5:   1. Title changed to: *Qualitative findings on the social relationships of DSRD caregivers.* 2. Impact on social networks changed to: *Impact on Social Relationships.* 3. Social network size changed to: *Perceived Shrinkage of Social World.* |
| Rename the outcome construct entirely | Thank you for this comment. As noted above, the verbiage changes have necessitated changes to the outcome constructs which are now reflected throughout the manuscript and are highlighted in the section above.  Specifically, the four main fields have been renamed: 1) Quality of Adult Friendships, 2) Impact on Social Relationships, 3) Spouse/Partner Impact, and 4) Perceived Shrinkage of Social World. The sub-sections of these have also been modified in the Impact on Social Relationships (Decreased Social Contacts), and Perceived Shrinkage of Social World (Reduced Invitations from Social Contacts, Shift to Condition-Specific Support Relationships, and Perceived Enduring Loss of Social Contacts).  This is most clearly reflected in Table 1. In addition, Figures 1, 2, and 3, have been updated to reflect this as well. |
| Explicitly state: This is a narrative burden-of-care study, not a network study. | Thank you for this comment. The authors have made the suggested edits, verbatim, as follows:  Abstract:  *“This is a narrative burden-of-care study, not a network study. Using cross-sectional study design, caregivers of individuals with DSRD (n=228) and DSN (n=137) were recruited from a neurology clinic and a DSRD Facebook support group.”*  Methods:  “*This is a narrative burden-of-care study, not a network study and utilized a cross-sectional study design*.” |
